# Tight Junctions as a Key for Pathogens Invasion in Intestinal Epithelial Cells

**DOI:** 10.3390/ijms22052506

**Published:** 2021-03-02

**Authors:** Tracy Paradis, Hervé Bègue, Louise Basmaciyan, Frédéric Dalle, Fabienne Bon

**Affiliations:** 1 UMR PAM l’Université de Bourgogne Franche-Comté (UBFC), AgroSup Dijon, Equipe Vin, Aliment, Microbiologie, Stress, F21000 Dijon, France; Tracy.Paradis@u-bourgogne.fr (T.P.); Herve.Begue@u-bourgogne.fr (H.B.); Louise.Basmaciyan@u-bourgogne.fr (L.B.); Fabienne.Bon@u-bourgogne.fr (F.B.); 2 Laboratoire de Parasitologie-Mycologie, Plateforme de Biologie Hospitalo-Universitaire Gérard Mack, F21000 Dijon, France

**Keywords:** enterocytes, gut barrier, tight junction, intestinal epithelial cells, pathogens, microorganisms, signaling pathways, permeability

## Abstract

Tight junctions play a major role in maintaining the integrity and impermeability of the intestinal barrier. As such, they act as an ideal target for pathogens to promote their translocation through the intestinal mucosa and invade their host. Different strategies are used by pathogens, aimed at directly destabilizing the junctional network or modulating the different signaling pathways involved in the modulation of these junctions. After a brief presentation of the organization and modulation of tight junctions, we provide the state of the art of the molecular mechanisms leading to permeability breakdown of the gut barrier as a consequence of tight junctions’ attack by pathogens, including bacteria, viruses, fungi, and parasites.

## 1. Introduction

A large community of microorganisms (viruses, bacteria, and fungi) and parasites inhabits the intestinal lumen forming the intestinal microbiota. This microbiota includes commensal microbial species that may, for some, become pathogenic following a disruption of the host defenses. The intestinal epithelial barrier segregates microorganisms and/or their components in the gut lumen considered as an external environment from the sterile deep tissues. The separation is achieved in part by intercellular junctions, especially tight junctions (TJ), which ensure impermeability of the gut barrier, avoiding the translocation of both commensal and pathogenic agents [1,2]. Commensal microorganisms have a protective role for the digestive mucosa by participating in maintaining the physiological integrity of TJs which sustain their barrier function during entero-pathogen challenge [3,4,5,6,7,8]. Among them, probiotics have been reported to potentiate the tightening of TJs, which improve impermeability of the gut mucosa aimed at, among others, counteracting the deleterious action of pathogens upon the TJ complex. In contrast, some pathogenic microorganisms have developed strategies to disorganize the TJs aiming to translocate through the digestive mucosa and invade their host. These entero-pathogens include (i) entero-invasive or entero-toxigenic bacteria (respectively, *Shigella, Salmonella*, enteroinvasive/enteropathogenic *Escherichia coli*, *Yersinia, Campylobacter,* or *Vibrio cholerae*, enterotoxigenic or hemorrhagic *Escherichia coli*, *Clostridium,* and *Aeromonas*), (ii) enteric viruses that are specific to the digestive tract, such as *Norovirus*, *Astrovirus,* and *Rotavirus* but also viruses such as HIV that can target enterocyte TJs, (iii) parasites causing gastroenteritis (i.e., *Entamoeba, Blastocystis,* or *Giardia*), or using intestinal cells as a portal of entry for their dissemination (i.e., *Toxoplasma gondii*), and (iv) fungi, including the yeast *Candida albicans* belonging to the gut mycobiote, and other fungi such as *Aspergillus* and *Penicillium* that are transitory hosts of the food bolus.

**Figure 1:**
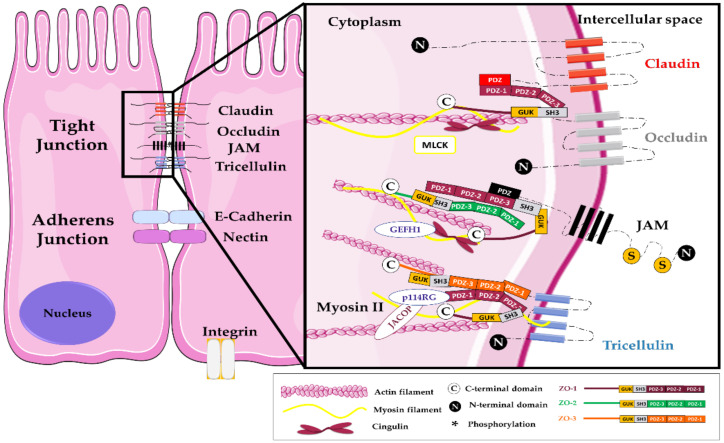
Composition of tight junctions in intestinal epithelial cells. Among the over forty proteins involved in the TJ complex, the transmembrane proteins belong mainly to three groups: the claudins family, the Marvel domain-containing proteins (occludin, tricellulin, also known as MarvelD2 and MarvelD3 proteins), and several immunoglobulin superfamily members ((Junctional Adhesion Molecules (JAMs), Coxsackie and Adenovirus Receptor proteins (CAR)) [17]. Other transmembrane proteins such as BVES (blood vessel epicardial substance), the apical polarity determinant Crb3 (Crumbs Cell Polarity Complex Component 3), and angulins colocalize and interact with TJs at the apical level even if not consensually considered to belong to this junctional complex [18]. These proteins relate to a cytoplasmic plaque formed by adaptor and signaling proteins. Adaptor proteins provide a bridge to the cytoskeleton through actin and microtubule tight bonds and include proteins such as zonula occludens proteins (ZO-1/3), cingulin, paracingulin, also known as junction-associated-coiled-coil protein (JACOP), membrane-associated guanylate kinases (MAGI 1–3), Multi-PDZ domain protein 1 (MUPP1), Pals, PATJ, Partitioning defective 3 and 6 proteins (Par3 and 6), Merlin–angiomotin complex. Signaling proteins complete this complex network, including protein kinases (atypical protein kinase C (aPKC), Mitogen-activated protein kinase kinase (MEKK1), complex Cyclin D1/CDK4, Large tumor suppressor kinase 1 (LATS1)), phosphatases (Phosphatase and TENsin homolog (PTEN)), GTPases and their activators (Rap2c, Myosin-IXA, Rho guanine nucleotide exchange factors (PDZGEF1, p114RhoGEF, GEF-H1, ARHGEF11), RICH1, SH3 domain-binding protein 1 (SH3BP1), Tubulin alpha chain (Tuba)), heat shock proteins (Apg-2), and transcriptional and post-transcriptional regulators ((zonula occludens 1-associated nucleic acid binding protein (ZONAB), Symplekin, yes-associated protein 1 (YAP), tafazzin (TAZ)). The last group of proteins localize with the TJ scaffold proteins but move to the nucleus aimed at modulating gene expression. Therefore, they are involved not only in the regulation of the junctional organization and function but also take part in many cells signaling pathways including cellular proliferation, differentiation, and response to many stimuli.

The cohesion and impermeability of the intestinal epithelium is based on the presence of junctional intercellular complexes formed at the apical level by TJs, adherens junctions (AJs), and desmosomes, all of them interacting with the cytoskeleton (Figure 1) [9,10]. Among them, TJs are involved in cell–cell interactions between enterocytes as well as with other intestinal cells, including intraepithelial lymphocytes, M cells, goblet cells, or dendritic cells. Each of them is associated with specific features and role in the barrier and chemical functions of the intestinal mucosa [11]. TJs form a continuous and tight branched network between membranes of neighboring cells, leading to the complete sealing of the apical intercellular space (Figure 1). In addition, TJ proteins, especially claudins, constitute a barrier inside the membrane itself, preventing the migration of transmembrane proteins and lipids from the apical to the baso-lateral side, therefore participating in enterocyte polarization [12]. Thus, TJs also act as a site for integration and transmission of signals necessary for the regulation of their assembly and for cell polarization. Finally, they participate to the modulation of gene expression required for cell proliferation and differentiation, as well as in stress responses [12,13,14,15,16]. Altogether, the TJ complex consists of a rate-limiting factor in the paracellular permeability, in response to environmental changes which undergo regulations of the integrity of the gut barrier through their opening or sealing. In this context, having a clear view of the mechanisms involved in TJ dynamics is crucial to understand their key role in preventing microbial translocation and spread to deep tissues.

After a brief presentation of the organization and modulation of TJs, this review will focus on the abilities of pathogens to target tight junctions with the aim to promote invasion of the gut mucosa and dissemination into the host.

## 2. Structure, Formation, and Modulation of Tight Junctions

The TJ complex is composed by three family of transmembrane proteins (i.e., the claudin family, the Marvel domain-containing proteins (occludin, tricellulin, and MarvelD3), and immunoglobulin superfamily (JAM, CAR)). This transmembrane structure is bounding to the cytoskeleton through a cytoplasmic TJ plaque composed of scaffold proteins among them the zonula occludens (ZO) proteins 1 to 3, the cingulin and cingulin-like proteins, and the afadine. Many other proteins involved in signal transduction and membrane trafficking are also associated with TJs (Figure 1).

The Tj formation initiates at apical pole of neighboring immature epithelial cells. The concomitant maturation of both tight and adherens junctions requires the activation of several signaling pathways, namely (i) the complex Par3-Par6-aPKC involving the partitioning defective homolog proteins 3 and 6 (Par-3) (Par-6) and the atypical protein kinase C (aPKC), (ii) several phosphatases (e.g., phosphatase 2A (PP2A)), and (iii) various GTPases (e.g., Raps, Rho GTPases, including RhoA, Rac, and Cdc42) [19,20,21,22,23]. The junctions between epithelial cells are considered functional when TJs and AJs are distinct [12].

**Figure 2:**
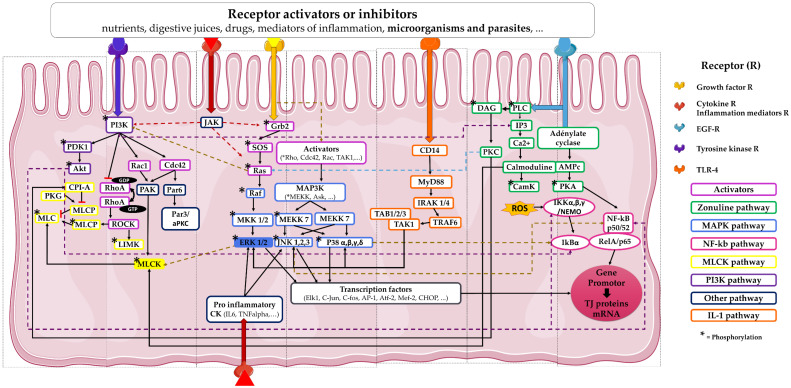
Signaling pathways involved in TJ formation and modulation. This scheme presents a non-exhaustive list of the different signaling pathways involved in the modulation of tight junctions of intestinal epithelial cells. Cytokines (CK). Janus kinase (JAK). Phosphoinositide-3-kinase (PI3K) and Myosin light chain kinase (MLCK) pathways: Phosphoinositide-dependent kinase-1 (PDK1), protein kinase B (Akt1), protein kinase G (PKG), Myosin light chain (MLC), Myosin light chain phosphatase (MLCP), LIM-kinase (LIMK), Ras homolog family member A (RhoA), Rho-associated coiled-coil containing protein kinase (ROCK), Ras-related C3 botulinum toxin substrate 1 (Rac1), Cell division control protein 42 homolog (Cdc42), p21-activated kinase 1 (PAK1), Partitioning defective 6 protein (Par6), Partitioning defective 3 protein (Par3), atypical protein kinase C (aPKC). Growth factor receptor-bound protein 2 (Grb2)/Mitogen-activated protein kinases (MAPK) pathway: Son of sevenless protein (SOS), Ras GTPase (Ras), Rapidly Accelerated Fibrosarcoma kinase (Raf), Mitogen-activated protein kinase kinases (MEKK, MKK), Extracellular signal-regulated kinase (ERK), c-Jun N-terminal kinase (JNK), Apoptosis signal-regulating kinase (Ask). IL-1 pathway: Myeloid differentiation primary response 88 (MYD88), interleukin-1 receptor-associated kinase (IRAK), Mitogen-activated protein kinase kinase kinase 7 (MAP3K7, also known as TAK1), TNF receptor-associated factor 6 (TRAF6), TGF-Beta Activated Kinase (TAB). Zonulin pathway: phospholipase C (PLC), Inositol trisphosphate (IP3), diacyl glycerol (DAG), protein kinase C (PKC), protein kinase A (PKA), Ca^2+^/calmodulin-dependent protein kinase (CamK), Reactive Oxygen Species (ROS), nuclear factor-kappa B (NF-κB), nuclear factor of kappa light polypeptide gene enhancer in B-cells inhibitor alpha (IκBα), I-κB-kinase (IKK), NF-κB Essential Modulator (NEMO), REL Proto-Oncogene, NF-κB Subunit (Rel). Tumor Necrosis Factor (TNF), Eph-like kinase 1 (Elk1), Activator protein 1 (AP-1), myocyte enhancer factor-2 (Mef-2), CCAAT-enhancer-binding protein homologous protein (CHOP).

TJ formation and dissociation are dynamic processes contributing to the modulation of tissue permeability in response to (i) variations in the chemical composition of the intestinal bolus (i.e., type and quantity of proteolytic enzymes, ionic content, and solutes), (ii) the inflammatory state of the intestinal mucosa, and (iii) the gut microbiota composition [24]. These are ensured by the development of vesicular trafficking of TJ proteins between the cytosol and the cell membrane, leading to their destruction and/or their rewiring to the cytosol, that correlates with their degree and sites of phosphorylation. Both changes in their expression and post-transcriptional regulatory mechanisms have also been reported but partially elucidated (Figure 2) [25]. Various exogenous factors and physiological modulators regulate the integrity of the TJs and consequently the intestinal permeability. In this context, the maintenance of the TJ proteins to the junctional complex has been correlated to the activation of kinase pathways including PKC and MAPK (ERK, p38, JNK), the calcium/calmodulin-dependent kinase 2 (CaMKK2)–AMP-activated protein kinase (AMPK), as well as Rho and NF-κB pathways [25,26,27,28,29,30,31]. Currently, zonulin (haptoglobin 2 precursor) remains the only endogenous modulator described as specific for TJs. It was identified as the mammalian analogue of zonula occludens toxin (Zot), secreted by the cholera pathogen *Vibrio cholerae* [32]. Whereas the secreting cell type remains to be specified, zonulin is produced in the intestinal lumen in response to the luminal presence of gluten and/or during bacterial colonization. In fact, its expression is associated with the presence of both commensal and pathogenic bacteria, with a more intense response to the latter [33,34]. Zonulin is able to activate the EGF receptor through direct binding or through the transactivation of the protease-activated receptor 2 (PAR_2_). This activation is then followed by the activation of the Ras-MAP-kinase cascade, ending by the removal of ZO-1 proteins from the junctional complex and therefore promoting the relaxation of tight junctions [34,35].

In polarized cells, the peri junctional contractile ring of actin–myosin II intimately interacts with TJ proteins. Consequently, any modification in one of these two connected compartments directly impacts physically the functional organization of the other. For instance, the actin–myosin contraction following the activation of the myosin light-chain kinase (MLCK) mediates intestinal TJ regulation in response to both physiological and pathophysiological stimuli [36]. In the same way, Rho proteins have been suggested to play an important role in maintaining the association of TJs with the membrane [37,38]. Indeed, the kinase ROCK, downstream effector of Rho, is reported to regulate specifically TJs via its effects on the F-actin cytoskeleton [38]. Besides, activation of the EGF receptor, leading to the activation of both β and ε isoforms of the protein kinase C (PKC), as well as the phospholipase C, promotes the stabilization of the actin/myosin ring and consequently the TJ complex [39]. To our knowledge, signaling pathways associated to TJ modulation have been reviewed (i) concomitantly in different cellular models other than intestinal epithelial cells (IECs) or (ii) singly or in a specific context in IECs [40,41,42,43,44,45].

## 3. Modulation of Tight Junctions by Pathogenic Microbial Agents

TJs constitute the backbone of the first line of the host defenses by limiting pathogen intrusion from the intestinal lumen to the sterile underlying tissues. Hence, enteropathogenic microbial agents have developed various stratagems to weaken the gut epithelia, exploiting TJ components to either invade the cells and tissues or promote host and microbial signaling responses that potentiate their invasion [9,46,47,48]. In both in vivo and in vitro models of enteric infections (i.e., viral, bacterial, fungal, or parasitic), pathogen invasion has been reported to correlate with an increase of the intestine permeability. Table 1 lists studies that correlated the breakdown of gut barrier with a quantitative decrease of one or more proteins belonging to the TJ complex but without specifying the involved mechanisms.

Studies, listed here, mainly reported TJ protein fitness based on Western blotting and microscopy analyses during IEC infection without including involved mechanism characterization. Different outcomes of TJ proteins were reported: modification of cell distribution (L), modification of gene expression (E), variation of protein quantity (Q), and dissociation +/− degradation of the TJ complex (D). Enteroaggregative *Escherichia coli* (EAEC), Enterohemorrhagic *Escherichia coli* (EHEC), Enteropathogenic *Escherichia coli* (EPEC), Enterotoxigenic *Escherichia coli* (ETEC). In the gut, TJ modulation occurs through specific microbial effector molecules that are bound to their membrane, capsid, or cell wall, or secreted in the intestinal lumen, or, for gram negative bacteria, injected into the cytoplasm of the host cells using type III secretion systems (T3SS) [88,89,116,117,118]. These effectors can act directly upon constitutive TJ proteins through either their lytic activity (i.e., proteases, lipases, phosphatases) leading to the degradation of TJs or through specific binding allowing the disengagement of TJ proteins from the junctional complex (Table 1). Pathogenic factors can also trigger cell signaling pathways involved in both TJs and cytoskeleton modulation by either inducing up- or down-regulation of gene expression or post-transcriptional events such as phosphorylation. Finally, pro-inflammatory and/or oxidative stresses of IECs resulting from infection can potentiate dysregulation of the TJ complex.

**Figure 3:**
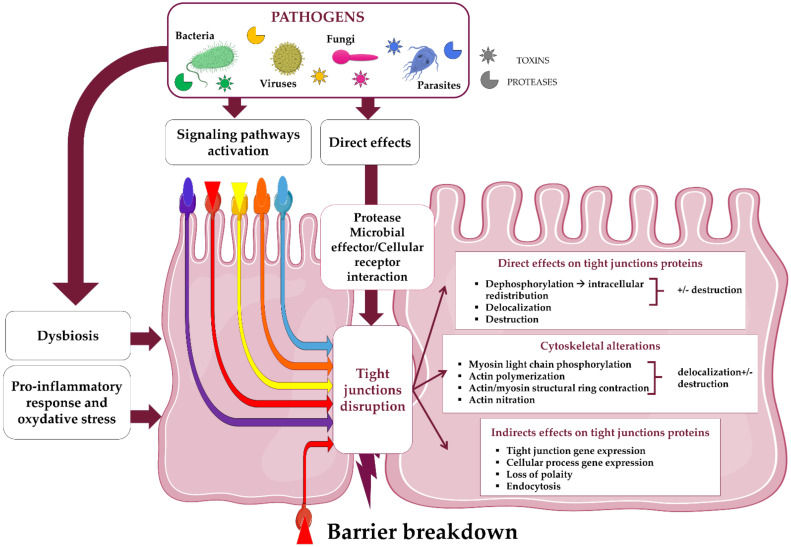
Role of tight junction interactions in pathogenesis of microorganisms. The compromise of TJ integrity involved different strategies developed by microbial pathogens. Microorganisms or their components directly impact the TJ organization through lytic activity or binding on TJ proteins that interfere on their connection to the TJ complex. Interactions between microbial effectors with IEC receptors lead in the activation of different signaling pathways intimately linked and interacting with each other. As these pathways are involved in many other cellular process (proliferation, oncogenesis, cell cycle, differentiation…), their activation contributes to the host response concomitantly to TJ disruption. Besides, the loss of permeability related to TJ appears as a consequence of a deleterious host response against its aggression though excessive pro-inflammatory cytokine production and endogenous biosynthesis of nitric oxide (NO).

In the following sections, we provide an overview of the different microbial strategies aimed at invading the digestive mucosa by targeting the TJ complex (Figure 3 and Table 2).

### 3.1. Action of Microbial Toxins on the IECs’ Cytoskeleton

Some microorganisms use effector toxins that destabilize the architecture of the cytoskeleton or promote contraction of the myosin/actin ring, through the activation of MLCK or Rho GTPases pathways, both inducing a subsequent TJ disruption [49,50,51,132,133,134,135].

Viral enterotoxins induce the rearrangement of F-actin filaments and/or microtubules, leading to TJ disruption as exemplified with enteric viruses such as *Astrovirus* or *Rotavirus* [49,50,51]. The mechanisms involved in permeability increase during these viral infections has not yet been elucidated.

In bacteria, the glucosyltransferase activity of the secreted toxins A and B of *Clostridium difficile* activates both isoforms α and β of the cellular kinase PKC, leading to the RhoA glycosylation in T84 IECs [136]. The resulting inactivation of the Rho GTPases allows actin rearrangement and the dissociation of the actine/ZO-1 complex, followed by the subsequent remove of occludin, ZO-1 and 2 from the junctional plaque [37,134]. During *Salmonella* infections, the pathogenesis process is mainly associated with the injection of the secreted proteins SopB, SopE, SopE2, and SipA in the host cell cytoplasm, that also activate Rho GTPase and PKC pathways [116,117]. Furthermore, the entero-pathogenic *Escherichia coli* (EPEC) has been reported to induce actin/myosin ring contraction and subsequent TJ disruption through calcium- and MLCK-dependent processes triggered by injected T3SS factors (i.e., Tir, EspB, EspF, EspH, and Mad) [88,89,118]. Another example of bacterial toxin targeting the actin/myosin ring has been reported for the zonula occludens toxin (Zot) of *Vibrio cholerae* that acts by activating the EGF/PAR_2_ receptor, leading to a cascade of phosphorylation initiated by phospholipase C and then PKC [33,34,137,138,139]. In fine, this results in the contraction of the actin/myosin ring associated with the displacement of the junctional complex ZO-1/ZO-2 from the membrane to the cytosol [34].

Similar approaches have been observed in fungi belonging to *Aspergillus* and *Penicillium* genera [140]. For instance, fungal metabolites such as cytochalasin D or the mycotoxin patulin have been reported to activate the MLCK regulatory pathway, leading to the disruption of F-actin filaments or the inhibition of its polymerization. Subsequently, the TJ organization is impaired along with cellular processes such as cellular endocytosis [24,127].

### 3.2. Direct Interaction of the Microbial Agents with TJ Proteins

Pathogens can target TJ proteins using virulence factors either localized on the pathogen outermost layer (i.e., cell wall or capsid/envelop), or secreted directly or through vesicles transported in the intestinal lumen. In this context, two major mechanisms have been observed: (i) a disorganization of the TJ network involving microbial components displaying lipase or protease activities on TJ complex or (ii) a direct interaction of microbial effectors with one or several TJ protein(s), leading to its/their detachment or relocation from the junctional complex to other cellular compartments.

Many pathogenic agents secrete enterotoxins exhibiting a protease activity, as reported for the fragylisin enterotoxin (BFT) of *Bacteroïdes fragilis*, the aerolysin of *Aeromonas hydrophila*, the hemagglutinin protease (Ha/P) of *Vibrio cholerae* and the serine protease High temperature requirement protein A (HtrA) of *Campylobacter jejuni* [52,53,54,55,56,118,120,121,122,134,141,142,143].

Whether they are secreted or not, microbial effectors can directly interact specifically with host TJ proteins. Thereby, the *Clostridium perfringens* enterotoxin (CPE) binds to claudins 4 leading to its cytosolic localization and destruction [90]. Similarly, during digestive amebiasis, interaction of the cysteine proteinase rEhCP112 of *Entamoeba histolytica* with claudins 1 and 2 leads to their degradation and cytosolic localization [130]. In the same way, *Toxoplasma gondii* co-localizes with the extracellular loops of the occludin during invasion of IECs. This physical interaction induces changes in the distribution and partitioning of occludin [57]. However, the molecular mechanism driving these events as well as the parasite effector molecules involved remain to be clarified.

### 3.3. Microbial Modulation of Signaling Pathways Involved in TJ Organization

Microbial agents can modulate signaling pathways involved in the structural organization of the TJ complex, leading to (i) changes in gene expression encoding TJ proteins or (ii) post-transcriptional events (e.g., level and site of phosphorylation of TJ proteins, protein trafficking) involved in the localization and partitioning of the proteins from the TJs to the cytoplasm (Table 2). In fine, all these events favor an increase in IECs’ permeability.

As specified in Section 3.1, the MLCK and Rho signaling pathways are key pathways for TJ formation and modulation also in various models of infection with the aim to increase gut permeability (Table 2).

The PKA signaling pathway has been reported as a possible target for enteric pathogens that consequently increase gut permeability. For instance, binding of the enteropathogenic *Escherichia coli* (EPEC) heat-stable enterotoxin A (STa) to the extracellular domains of the guanylate cyclase receptor (GC-C) catalyzes cGMP formation. This complex further activates the cAMP-dependent-protein kinase A (PKA) leading to, among others, a dephosphorylation of the occludin protein and its subsequent redistribution from the membrane to the cytosol [142,144,145,146]. Similarly, during *Rotavirus* infection of IECs, a specific decrease in the occludin protein level at the TJ complex is reported as the result of the downregulation of occludin gene transcription with regulatory signals involving both Rp-cyclic AMP and PKA pathways [58].

Microbial components through the modulation of the MAPK pathway can alter TJ organization (Table 2). During *Shigella flexneri* or EPEC infection, the dysfunction of the gut barrier results from changes in the level of phosphorylation of claudin-2 and -4, occludin and ZO-1 proteins, through ERK1/2 pathway [59,91]. However, other kinases belonging to the MAPK pathway may be specifically involved in bacterial TJ alteration, as observed in a *Yersinia enterolitica* model of IECs infection. In this model, the decrease in claudin 8 levels correlates with the phosphorylation of JNK but not of the other MAP kinases [92].

Signaling pathways are diverse and numerous. They notably act jointly in TJ regulation, some of them probably being concomitantly targeted by probiotics to prevent TJ alteration induced by pathogens (Table 3). However, in most of the studies investigating modulation of the TJ complex by pathogens, the sequence of the signaling cascade is partially investigated, leaving numerous gaps in our knowledge of the precise mechanisms involved in these modulatory events. Nevertheless, the molecular mechanisms drawing the breakdown of the intestinal barrier associated with TJ alteration have been better characterized in few infection models.

Thereby, the example of the action of *zonula occludens* toxin (Zot) from *Vibrio cholerae* upon IECs’ permeability highlights the complexity of the TJ regulatory network involved during microbial infection. The synthetic peptide AT-1002, corresponding to the C-terminal domain of Zot, induces alteration of TJs. This results from the activation of the MAPK pathway (JNK) which ultimately leads to the phosphorylation of the tyrosine residues of ZO-1 and its remove from the TJ plaque together with F-actin rearrangement [125,153]. Besides, the phospholipase C/PKC pathway is also involved as suggested by the binding of its associated EGF/PAR_2_ receptor to Zonulin, a eukaryotic analogue of Zot. Its activation ends with an increase in the intracellular calcium concentration, a further contraction of the actin/myosin ring and finally the shifting of the junctional complex ZO-1/ZO-2 from the membrane to the cytosol [33,34,139]. In addition, other bacterial toxins, including the heat-stable toxin b (Hst b) from enterotoxigenic *Escherichia coli* or the Zot from *Campylobacter concisus*, display partial sequence homology with the active domain of Zot from *Vibrio* and potentially share regulatory pathways involved in modulating TJ complex [60,107].

Another example of the cooperation of several pathogen-targeted cellular pathways has been described during IECs treatment with patulin. As presented above, this mycotoxin activates the MLCK regulatory pathway. In parallel, it decreases expression of both the density-enhanced phosphatase-1 (DEP-1) and Peroxisome ProliferAtor Receptor gamma (PPARγ), the latter controlling DEP-1 expression [93]. The subsequent hyper-phosphorylation of claudin 4 protein then induces disturbances in claudin-4/ZO-1 interactions, hence favoring their release from the TJ complex [93].

Many viruses, including *Adenovirus*, *Coxsackievirus*, Hepatitis C virus, and *Rotavirus,* have been reported to target extracellular domains of the TJ constitutive proteins [46]. The cellular receptor within the enterocyte TJ complex has been indeed identified for at least three viruses including (i) *Rotavirus* attaching to the JAM protein and (ii) *Coxsackievirus* and *Adenovirus* that bind to *Coxsackievirus* and *Adenovirus* receptor (CAR), a transmembrane protein associated with ZO-1 either directly or through intermediary proteins, namely MAGI-1 and MUPP1 [106,154]. However, the following events promoting the disorganization of TJs and the resulting decrease in IECs’ permeability remain to be specified, except for *Coxsackievirus* B for which molecular mechanisms have been detailed following its binding to CAR. Indeed, during IECs’ infection, *Coxsackievirus* B does not induce major TJ reorganization, but stimulates the specific internalization of occludin within macropinosomes [61]. Concomitantly, this virus interacts with the intestinal epithelial protein DAF (Decay-Accelerating Factor) leading to its redistribution into lipid rafts. This DAF localization is followed by the successive activation of the tyrosine kinase c-Ab1 and the Rho GTPases Rac. The subsequent actin remodeling allows the transport of DAF bound particles into TJ complex. All these events are required for the viral entry. [61,62,108].

Finally, most of the pathways cited here are not restricted to a single cellular process. Indeed, for instance, activation of the MLCK pathway promotes an increase in IECs’ permeability, as a consequence of both the modulation of the TJ structure and intracellular Ca^2+^ influx. This was exemplified with the bacterial aerolysin from *Aeromonas hydrophila* that has been shown to target the MLCK signaling cascade, promoting the disassembly of claudins 1 and 4 concomitantly to an increase in the intracellular influx of Ca^2+^, both mechanisms being necessary for disorganization of the TJ complex and the subsequent decrease in IEC permeability [54].

## 4. Indirect Modulation of the TJs Consequently to the Host Response Facing Infection

Finally, the modulation of intestinal TJ integrity can also result from IECs deleterious inflammatory response facing the infection process. Altered intestinal TJ integrity has been reported to result from pro-inflammatory events involving cytokines such as IL-1β, IL-4, IL-6, IL-13, and TNF-α/IFNγ secreted by IECs, that subsequently activate regulatory pathways linked to the TJ complex [24,155,156,157]. Some of these cytokines, including IL-1β and IL-18, are secreted following activation of the inflammasome pathway during IECs’ infection [158].

Focusing on the IL-18 pathway, during IECs’ infection by HIV-1, the transactivator HIV Tat protein binds to TLR4-MD2-CD14 epithelial complex, activating the NF-κB pathway. Consequently, mature IL-18 is released by IECs that subsequently induces an increase of permeability by (i) decreasing and disrupting both TJs and AJs and (ii) altering cytoskeleton. In parallel, the treatment of the IECs with IL-18 decrease the expression of both claudin-2 and occludin as determined by Western blot analyses. These observations correlate with an increase in the expression of MLCK and phosphorylation of MLC by ROCK [119].

Regarding the IL-6 pathway, interaction of the secreted *Listeria* adhesion protein (Lap) to the host cell Hsp60 receptor promotes activation of the NF-κB pathway [63]. This activation induces the MLCK-mediated opening of the TJs, associated with a concomitant upregulation of pro-inflammatory TNFα and IL-6 [63]. These observations suggest that the disorganization of the TJ complex may associate an inflammatory burst that will secondarily accentuate the decrease in IECs’ permeability. For instance, the release of IL6 by IEC induced by LPS has been reported to decrease membrane integrity [159,160]. Moreover, interestingly, in Gram-negative bacterial infection models, the release of lipopolysaccharide (LPS) has been shown to decrease the transcriptional expression of occludin and a cytosolic localization of claudin 1 [161]. However, LPS-induced secretion of IL-6 upon TJ integrity has not been investigated.

During interaction of IEC with live or heat-killed *Candida albicans* cells, the decrease of gene expression of ZO-1 and occludin is correlated with an inhibition of the NLRP3/NLRP6 inflammasome expression, suggesting that the loss of TJ integrity occurs independently of a direct *C. albicans* activity [64].

Furthermore, in response to infections, endogenous biosynthesis of nitric oxide (NO) regulates IECs functionality both directly (through free radical activity) and indirectly through cell signaling mechanisms that impact tight junction protein expression, including the PKC, MAPK (ERK, p38, JNK), Rho, and NF-kb pathways [41]. The subsequent TJ disruption and epithelial damages favor the intestinal translocation of microbial pathogens [41,162]. This was exemplified in IPEC-J2 exposure to fungal secreted ochratoxin, which induces ROS generation associated with an increase in intracellular Ca^2+^ concentrations [65]. The subsequent activation of the MLCK pathway finally leads to disruption of the TJs associated with an increase in IECs’ permeability [65]. Ochratoxin has been shown to also increase Caco-2 epithelial permeability by promoting the remove of ZO-1 and claudin-1 from the TJ complex [94].

## 5. Conclusions

In conclusion, some enteric pathogens can target the junctional complex to weaken the intestinal epithelial barrier and promote their invasion. This microbial modulation of the IECs’ permeability involves numerous mechanisms ranging from direct molecular interactions of microorganism with host components to the modulation of various signaling cellular pathways. Whereas many studies highlight the fate of the major TJ proteins (i.e., occludin, claudins and ZO-1) during bacterial, viral, fungal, or parasitic IEC infections, the cellular and molecular mechanisms remain to be specified, including the nature of the microbial effector, its host cellular receptor, the nature of the signaling pathways involved, as well as the direct or indirect impact of their modulation on the TJ complex organization. Finally, most of the observations reported here are based on simplified models of infection (one pathogen interacting with one type of intestinal cell) that do not consider (i) the complexity of the intestinal ecosystem and environmental conditions including the communications between pathogenic and commensal microorganisms, or (ii) the influence of the intestinal microbiota upon pathogens/epithelial cells interactions regarding the gut TJ integrity [163,164,165]. Pathogenic effectors and/or their cellular receptors constitute therapeutic candidates by preventing the weakening of the digestive barrier induced by pathogens.

## Figures and Tables

**Table 1:** Main structural proteins of the tight junction complex in intestinal epithelial cells targeted by pathogens during their infection process.

TJ Proteins	Enteric Pathogens			Ref.
	Bacteria	Virus	Fungi and Parasites	
**Occludin**	*Aeromonas hydrophila* (D) *Campylobacter jejuni* (L/D) *Clostridium difficile* (L/D) *Clostridium perfringens* (L) *Escherichia coli*: *EAEC* (L), *EHEC* (L), *EPEC* (L/D), *ETEC* (L/D), K12 (E/Q) *Helicobacter pylori* (D) *Listeria monocytogenes* (L) *Salmonella typhimurium* (Q/L) *Staphylococcus aureus* (Q) *Shigella flexneri* (Q/L) *Vibrio cholerae* (D/L) *Yersinia enterolitica* (Q)	*Astrovirus* (L) *Coxsackievirus B* (L) *Norovirus* (Q) HIV-1 (E/Q) *Rotavirus* (E/Q/L)	*Anisakis simplex* (L) *Aspergillus, Penicillium* (D/Q/L) *Blastocystis* spp. ST17 (D) *Candida albicans* (Q) *Cryptosporidium parvum* (Q) *Giardia* spp. (L) *Toxoplasma gondii* (L)	[34,37,49,50,51,52,53,54,55,56,57,58,59,60,61,62,63,64,65,66,67,68,69,70,71,72,73,74,75,76,77,78,79,80,81,82,83,84,85,86]
**Tricellulin**	*EPEC* (Q) *Yersinia enterolitica* (Q)			[80,87]
**Claudin family**	*Aeromonas hydrophila* (D) *Campylobacter jejuni* (L) *Clostridium perfringens* (D/L) *Escherichia coli*: *EAEC* (L), *EHEC* (L/Q), *EPEC* (E/D/L), *ETEC* (D/L), K12 (E/Q/L) *Helicobacter pylori* (D/L) *Listeria monocytogenes* (L) *Salmonella typhimurium* (L) *Shigella flexneri* (Q/L) *Yersinia enterolitica* (Q/L)	*Astrovirus* (L) HIV-1 (E/Q) *Rotavirus* (L) *Norovirus* (Q)	*Aspergillus* and *Penicillium* (D/L Q) *Candida albicans* (Q) *Cryptosporidium parvum* (Q) *Entamoeba histolytica* (L) *Giardia* spp. (L)	[49,50,54,60,63,67,68,69,70,71,72,73,75,78,80,84,85,88,89,90,91,92,93,94,95,96,97,98,99,100,101,102,103,104,105]
**JAM-A**		*Rotavirus*	*Candida albicans* (Q)	[84,106]
**Zonula occludens 1–3**	*Aeromonas hydrophila* (D) *Clostridium difficile* (D/L) *Escherichia coli*: *EAEC* (L), *EHEC* (L), *EPEC* (E/D/L), *ETEC* (D/L), K12 (E/Q/L) *Helicobacter pylori* (L) *Salmonella typhimurium* (Q/L) *Staphylococcus aureus* (Q) *Shigella flexneri* (Q/L) *Vibrio cholerae* (L) *Yersinia enterolitica* (Q)	*Adenovirus* (L/Q) *Astrovirus* (L) *Coxsackievirus B* HIV-1 (E/L) *Rotavirus* (L)	*Anisakis simplex* (L) *Aspergillus* and *Penicillium* (D/Q/L) *Blastocystis* spp. ST17 (D/L) *Candida albicans* (Q) *Cryptosporidium andersoni* (L) *Cryptosporidium parvum* (Q) *Entamoeba histolytica* (D) *Giardia* spp. (D/Q/L)	[34,37,49,50,51,52,53,54,58,59,60,61,62,64,65,66,67,68,69,71,73,74,77,78,79,81,82,85,92,93,94,102,107,108,109,110,111,112,113,114,115]

**Table 2:** Examples of mechanisms involved in TJ modulation by pathogens that induces intestinal barrier breakdown permeability.

Pathogens	Host Cell Receptor (H) and/or Pathogens Elements (P) Involved	Activated Host Pathways in IEC	Junctions and Cytoskeleton Modeling	Ref.
Viruses				
*Adenovirus*	CAR cell receptor (H)	Nc	ZO-1 (p↓) (L)	[108]
*Astrovirus*	Capsid protein (P)	Nc	Occludin, claudin, ZO-1 (L) Actin rearrangement	[51]
*Coxsackievirus B*	Epithelial DAF (H) CAR cell receptor (H) Viral particles (P)	Rho GTPases	ZO-1 (p↓) (L) Occludin (L) Actin rearrangement	[61,62,108]
HIV-1	TLRA-MD2-CD14 (H), Tat protein (P)	NF-κB (IL-18) MLCK	Occludin, claudin (p↓) F-actin (p↓)	[119]
	gp120 (P)	Nc	Occludin, claudins 1-2, ZO-1 (p and g↓), ZO-1 (L)	[71]
*Rotavirus*	Nc	PKA	Occludin (g↓), phosphorylated form (p↓)	[58]
Bacteria				
*Aeromonas hydrophila*	Bacterial aerolysin (P)	Intracellular Ca^2+^ influx MLCK	Occludin, claudins 1/4/5, ZO-1 (L) F-actin condensation	[54]
*Bacteroïdes fragilis*	Fragilysin (P)	Nc	E-cadherin and F-actin disassembly	[120,121,122]
*Campylobacter concisus*	Zot toxin (P)	NK-κB Pro-inflammatory cytokines	ZO-1 (g↓) Cytoskeleton rearrangement	[107]
*Campylobacter jejuni*	Proteases (P)	None. Direct action	Occludin and E-cadherin (L)	[55]
	Serine protease HtrA (P)	None. Direct action	Occludin (L)	[56]
*Clostridium difficile*	Toxins A and B	Rho GTPases	Occludin, ZO-1/2, E-cadherin (L), Actin rearrangement	[37]
*Clostridium perfringens*	Nc	CpAL system	Occludin, claudin 3 (L)	[72]
	CPE Enterotoxin (P)	Binding to claudins 3/4	Claudin 4 (p↓) (L)	[90]
	Delta toxin (P)	ADMA10	E-cadherin (p↓)	[123]
*E. coli EAEC*	Nc	AAF/II action	Occludin, claudin1, ZO-1 (L)	[73]
*E. coli EHEC*	Shiga/vero toxins (P)	MLCK	Occludin, claudin 3, ZO-1 (L) Claudin 2 (p↓)	[74,75]
*E. coli EPEC*	EspG1 effector (P)	Nc	Tricellulin (p↓)	[87]
	EspF, EspI, EspG, Map, CNF-1, Tir effectors (P)	MLCK	Occludin, claudin 1 and ZO-1 (L) Actin-myosin ring contraction	[88,89,97,98,99,100]
	Extracellular vesicles and secreted factors (P)	Nc	Occludin, claudins, ZO-1/2 (g↓) Occludin, ZO-1 (L) F-actin rearrangement	[59]
*E. coli ETEC*	Stb toxin (P)	Nc	Occludin, claudin 1, ZO-1 (L)	[60]
*Helicobacter pylori*	VacA and CagA factors (P)	Nc	ZO-1 (L)	[109]
	Unspecified	MLCK	Occludin, claudins 4/5 (L)	[76]
	IL-R1 receptor (H) Bacteria contact (P/H)	ROCK activation	Claudins 1/4, ZO-1 (L)	[101,102]
*Listeria monocytogenes*	Hsp60 cell receptor (H) LAP protein (P)	NF-κB, MLCK Secretion TNFα IL6	Occludin, claudin 1, E-cadherin (L)	[63]
*Salmonella typhimurium*	SopB, SopE, SopE2, SipA factors (P)	Rho GTPase IL-8	Occludin, ZO-1 (L) Actin (L)	[66]
	Nc	PKC	ZO-1 and pZO-1 (p↓) Claudin 1, ZO-2 (L)	[77]
*Staphylococcus aureus*	Alpha toxin (P)	Nd	Occludin, ZO-1/3, E-cadherin (p↓)	[79]
*Shigella flexneri*	T3SS protein injection effector (P)	Nd	Occludin, p-occludin (p↓) Claudin 1 and ZO-1 (p↓) (L)	[67]
	SepA (P)	LIMK1 (g↓) Cofilin	Actin modification	[124]
	Nd	ERK1/2	Claudins 2/4 (L)	[91]
*Vibrio cholerae*	Hemagglutinin protease HA/P (P)	Nd	Occludin, ZO-1 (L) Actin rearrangement	[52,53]
	PAR_2_ receptor (H) Zot (P)	PLC PKC	Occludin, ZO-1 (L) Myosin phosphorylation Actin polymerization	[34,125]
*Yersinia enterolitica*	Nd	MAPK (JNK)	Claudins 2/3/8/10, ZO-1 (p↓), Claudins 3/4/8 (L)	[126]
Fungi and parasites				
*Aspergillus and Penicillium*	Ochratoxin (P)	MLCK ROS response [Ca^2+^]c increase	Claudin 1, ZO-1 (p↓) Occludin and ZO-1 (L)	[65,94]
	DEP-1 cellular protein (H) Patulin (Pat) (P)	DEP-1 (g↓) PPARγ protein (p↓), p-MLC-2 (p↑)	Occludin, ZO-1 (p↓) Claudin 4 (L)	[82,93,110,127]
*Blastocystis* spp.	Galactose residues on cell surface (H)	Nd	Occludin, ZO-1 (p↓)	[83]
	Cathepsin B (P)	ROCK p-MLC	ZO-1 (L)	[128,129]
*Candida albicans*	Nd	MAPK	Occludin, claudins 1/3/4, JAM-A (p↓)	[84]
	Heat-killed yeasts (P)	NLRP3/NLRP6	Occludin and ZO-1 (p/g↓)	[64]
*Entamoeba histolytica*	Cystein proteinases (P)	Nd	ZO-1 (p↓) ZO-1/ZO-2 (L)	[112]
	Secreted Prostaglandin E2 (P)	Nd	Claudin 4 (L)	[104]
	rEhCP112 proteinase (P)	Direct interaction	Claudins 1/2	[130]
*Giardia* spp.	Nc	MLCK	ZO-1 (L) Actin rearrangement	[86,105,113,114]
*Toxoplasma gondii*	Extracellular loops of occludin (H)	Direct interaction	Occludin (L)	[57,131]

Studies, listed here, were conducted only in enteric infection models of murine IEC (IEC-6, m-IC), porcine IEC (IPEC-J2) or human IEC (Caco-2, C2BBe1, HT29, HT29/B6, T84, HCT116, HCT-8) and gastric (NCI-N87 and their derived cell model HGE-20) epithelial cells. Protein modulation is documented with (L) when associated with localization change or with arrows illustrating protein cellular amount or gene expression increase or decrease respectively (p↑), (p↓), or (g↓). Nc: Non-characterized. AAF-II: Aggregation Adherence *Fimbriae* II, CAR: *Coxsackievirus* and *Adenovirus* Receptor, CpAl: *Clostridium perfringens* Arg-like system, DAF: Decay-Accelerating Factor, HA/P: Hemagglutinin Protease, htrA: high temperature requirement protein A, InlC: Internalin C, LAP: *Listeria* Adhesion Protein, MLCK: Myosin Light Chain Kinase, PAR2: Protease activated receptor 2, PKA: Protein Kinase A, Sop: *Salmonella* outer protein, SPI: *Salmonella* Pathogenicity Island1, Stb: *Escherichia coli* heat stable toxin b, Tcd: *Clostridium difficile* toxin, ZO: *Zonula occludens*, Zot: ZO toxin.

**Table 3:** Tight junction protection by probiotics preventing breakdown permeability induced during infections.

Probiotics	Pathogens	TJ Protein Modulation	Ref.
*Lactobacillus acidophilus*	*Salmonella typhimurium*	Modulation of 26 genes linked to TJ integrity	[147]
*Saccharomyces boulardii*	*Salmonella typhimurium*	Interference on Rho GTPase activation	[148]
*Escherichia coli Nissle 1917*	*Campylobacter jejuni*	Increase of gene expression of the claudins 2/4/11	[149]
*Lactobacillus reuteri* (LR1)	EPEC	Inactivation of MLCK pathway	[150]
*Lactobacillus plantarum* (GRI-2) *Lactobacillus rhamnosus* (LG6) *Lactobacillus fermentum* (FA-1) *Lactobacillus salivarius* (GPI-1)		Maintenance of membrane localization and gene expression of ZO-1, occludin, claudins 1/4 and JAM-A	[151]
*Bacillus subtilis* (CW14)	Fungal ochratoxin A	Prevention of ZO-1 destruction	[94]
*Bacillus clausii*	*Rotavirus*	Overexpression of occludin and ZO-1 proteins	[152]

## Data Availability

Not applicable.
